# On the Validity of Neural Mass Models

**DOI:** 10.3389/fncom.2020.581040

**Published:** 2021-01-05

**Authors:** Nicolás Deschle, Juan Ignacio Gossn, Prejaas Tewarie, Björn Schelter, Andreas Daffertshofer

**Affiliations:** ^1^Faculty of Behavioural and Movement Sciences, Amsterdam Movement Sciences & Institute for Brain and Behavior Amsterdam, Vrije Universiteit Amsterdam, Amsterdam, Netherlands; ^2^Institute for Complex Systems and Mathematical Biology, University of Aberdeen, King's College, Aberdeen, United Kingdom; ^3^Consejo Nacional de Investigaciones Científicas y Técnicas, Instituto de Astronomía y Física del Espacio, Universidad de Buenos Aires, Ciudad Universitaria, Buenos Aires, Argentina; ^4^Sir Peter Mansfield Imaging Centre, School of Physics and Astronomy, University of Nottingham, Nottingham, United Kingdom; ^5^Department of Clinical Neurophysiology and MEG Center, Amsterdam UMC, Vrije Universiteit Amsterdam, Amsterdam Neuroscience, Amsterdam, Netherlands

**Keywords:** neural mass model, leaky integrate and fire, random graph, mean field approximation, Freeman model

## Abstract

Modeling the dynamics of neural masses is a common approach in the study of neural populations. Various models have been proven useful to describe a plenitude of empirical observations including self-sustained local oscillations and patterns of distant synchronization. We discuss the extent to which mass models really resemble the mean dynamics of a neural population. In particular, we question the validity of neural mass models if the population under study comprises a mixture of excitatory and inhibitory neurons that are densely (inter-)connected. Starting from a network of noisy leaky integrate-and-fire neurons, we formulated two different population dynamics that both fall into the category of seminal Freeman neural mass models. The derivations contained several mean-field assumptions and time scale separation(s) between membrane and synapse dynamics. Our comparison of these neural mass models with the averaged dynamics of the population reveals bounds in the fraction of excitatory/inhibitory neuron as well as overall network degree for a mass model to provide adequate estimates. For substantial parameter ranges, our models fail to mimic the neural network's dynamics proper, be that in de-synchronized or in (high-frequency) synchronized states. Only around the onset of low-frequency synchronization our models provide proper estimates of the mean potential dynamics. While this shows their potential for, e.g., studying resting state dynamics obtained by encephalography with focus on the transition region, we must accept that predicting the more general dynamic outcome of a neural network via its mass dynamics requires great care.

## Introduction

Over the years, *neural mass models* have profoundly contributed to our understanding of the meso- and macroscopic dynamics of populations of neurons. This is particularly true when it comes to the oscillatory behavior of mean post-synaptic potentials and firing rates. Central there is the notion of brain rhythms arguably resembling (episodic) local and distant synchronization of neural oscillators. Corresponding theoretical studies date back as far as the mid of the last century (Beurle, 1956; Griffith, 1963, 1965) though it was Walter Freeman who coined the notion neural masses (Freeman, 1975). Building neural mass models typically relies on phenomenological insights and one “prescribes” the evolution of the neural activity over time (Deco et al., 2008). Yet, as said, these models strongly contributed to advancing our understanding of brain rhythms as they succeeded in mimicking signals recorded especially via magneto- and electroencephalography (Freeman, 1987; Kozma and Freeman, 2016).

Freeman's K-sets are based on a hierarchy of interacting sets of neural populations or masses (Freeman, 1975). These masses are composed of non-interacting, identical neurons. Without interactions in the mass, it is called a KO-set for which the original *Freeman model* applies. In fact, the absence of interactions in the mass allows for great algebraic simplicity: The mass dynamics can be cast in the form of a linear second-order ordinary differential equation,

(1) $$\begin{array}{l} \left[\frac{d}{dt}+\alpha \right]\left[\frac{d}{dt}+\beta \right]V=\left(\alpha \beta \right)J, \end{array}$$

where *V* = *V*(*t*) denotes the mean potential of all the somas over the neural population, *J* = *J*(*t*) represents some (common) input into the population, and α and β are (inverse) time constants specifying the rise and decay of the mean potential. The input *J* is usually a continuous function of time *t*, but if *J* is meant to resemble point processes, e.g., given via microscopic action potentials or spikes, continuity may be introduced (albeit heuristically) using sigmoidal activation functions (Marreiros et al., 2008).

Obviously, the dynamics (1) is a gross approximation for most (non-linear) neural dynamics at the microscopic level. Nonetheless, the model may provide reasonable approximations of selected features of a neural population's mean activity. However, apart from the weakness (if not absence) of internal interactions, this requires the population to contain sufficiently many neurons^1^ and/or symmetries. Symmetries typically imply an adequate amount of homogeneity. Even under these assumptions, detailed derivations of mass models like (1) from microscopic neural dynamics are rare (Stefanescu and Jirsa, 2008a; Byrne et al., 2017). As a consequence, it remains difficult to judge the degree to which the outcome of neural mass models really agrees with the mean activity of a “real” neural network. This is unfortunate because, given their mathematical ease, neural mass models appear ideal candidates for estimating parameter dependencies of network activation and predicting their dynamical outcome. For the Freeman model (1), Rodrigues and co-workers recently presented a mapping between a microscopic conductance-based model and the macroscopic mass dynamics (Rodrigues et al., 2010). They imposed strong homogeneity assumptions on a population of interconnected leaky integrate-and-fire (LIF) neurons. We adopted this approach but complemented it by an alternative, since some steps in Rodrigues et al. (2010) missed some rigor.

To test the quality of these two mass models, we simulated an externally driven, finite-size LIF network. We used its overall spiking activity and the external drive as input to the mass models and compared the resulting time series with the ones of the average LIF potentials using different measures. Following previous studies, we set these neural masses to contain neurons of mixed types, i.e., inhibitory and excitatory ones (Lopes da Silva et al., 1974; Jansen and Rit, 1995; Wendling et al., 2000; David and Friston, 2003; Stefanescu and Jirsa, 2008b; Ponten et al., 2010). Given the relevance for neural mass models in the study on brain rhythms we first investigated the spectral distributions of the potentials and complemented this by correlating the average network activity with that of the two neural mass models. This procedure allowed for a systematic assessment of the influence of population parameters on the quality of the mass model approximation(s). Here, we particularly focus on the (im-)balance between inhibitory and excitatory neurons and the degree of their connectivity. As will be shown, the quality of approximation(s) is (are) limited, except for parameter ranges defining the onset of synchrony and/or the range in which LIF neurons are synchronized at low spiking frequencies.

## Results

Our two neural mass models obey the generic form

(2) $$\begin{array}{l} \left[\tau^{\left(\text{mem}\right)}\frac{d}{dt}+1\right]\left[\tau^{\left(\text{syn}\right)}\frac{d}{dt}+1\right]V=\\ \;J^{\left(\text{net}\right)}\left(V;A,\ldots \right)+J^{\left(\text{ext}\right)}\left(V;\ldots \right) \end{array}$$

where *J*^(net)^(*V*; *A*, …) is the sum of all spike-related currents in the LIF network, i.e., the population mean of the expectation values of the spike trains generated by network times the corresponding synapse conductances. Its value may depend on the mean membrane potential *V* and the networks adjacency matrix *A* (amongst other parameters). *J*^(ext)^ summarizes the external drive, here always realized as a Poisson train. If the *V*-dependency of *J* is absent, we refer to (2) as the “conventional” Freeman model (CFM) and in the presence of a *V*-dependent currents, we refer to it as a slightly modified Freeman model (MFM); the explicit forms are given in (20) and (24), respectively.

The linearities in (2) stem from the facts that: (i) apart from the spike-related reset, the LIF dynamics is linear with membrane time constant τ^(mem)^; and (ii) we connected them via exponential synapses containing a linear conductance dynamics with time constant τ^(syn)^. In relation to (1) we have α = 1/τ^(mem)^, β = 1/τ^(syn)^.

In the absence of *J*^(net)^, the impulse response of (the left-hand side of) the dynamics (2) equals that of a second-order linear system with rise and decay times given by τ^(mem)^ and τ^(syn)^. The corresponding frequency response function is that of a second-order low-pass filter (cf. Appendix). As soon as *J*^(net)^ is included, i.e., once the LIF neurons start to fire, the response functions in the time- and frequency-domains become less trivial and quantitative assessments require numerical approaches. For this, we simulated a network composed of *N* = 10, 000 LIF neurons and used the simulated network's spiking activity as input to the mass dynamics, i.e., ~*J*^(net)^ in (2).

To study the influence of different adjacencies *A* on the quality of agreement between the average network potential and that modeled via the neural mass dynamics (2), we modified *A* in two ways. (i) We considered the adjacency of an Erdős-Rényi random graph and changed the network's overall degree *p* from 0 to 1 so *A* represents connections between all excitatory and inhibitory neurons. (ii) By the same token, we varied the relation between excitatory vs. inhibitory units that we quantified via

(3) $$\begin{array}{l} \lambda :=\frac{\#excitatory}{\#inhibitory+\#excitatory}. \end{array}$$

Details on the implementation of the different kinds of neurons can be found in the *Methods* section below; all parameters are summarized in Table 1.

**Table 1:** Parameters values used when simulating the network of LIF neurons.

**Variable**	**Description**	**Value**
*v*^(thres)^	Threshold potential	–50 mV
ṽ	Membrane reversal potential	–80 mV
ṽ^(*E*)^	Excitatory synaptic reversal potential	0 mV
ṽ^(*I*)^	Inhibitory synaptic reversal potential	–70 mV
*v*^(reset)^	Reset potential	–60 mV
τ^(mem)^	Membrane characteristic time	20 ms
τ^(*E*)^	Excitatory synaptic time constant	3 ms
τ^(*I*)^	Inhibitory synaptic time constant	7 ms
τ^(ref)^	Refractory period	5 ms
*g*	Leak conductance	10 nS
ĝ^(*E*)^	Excitatory synaptic conductivity	4 nS
ĝ^(*I*)^	Inhibitory synaptic conductivity	40 nS
ĝ^(ext)^	External synaptic conductivity	5 nS

**Figure 1:**
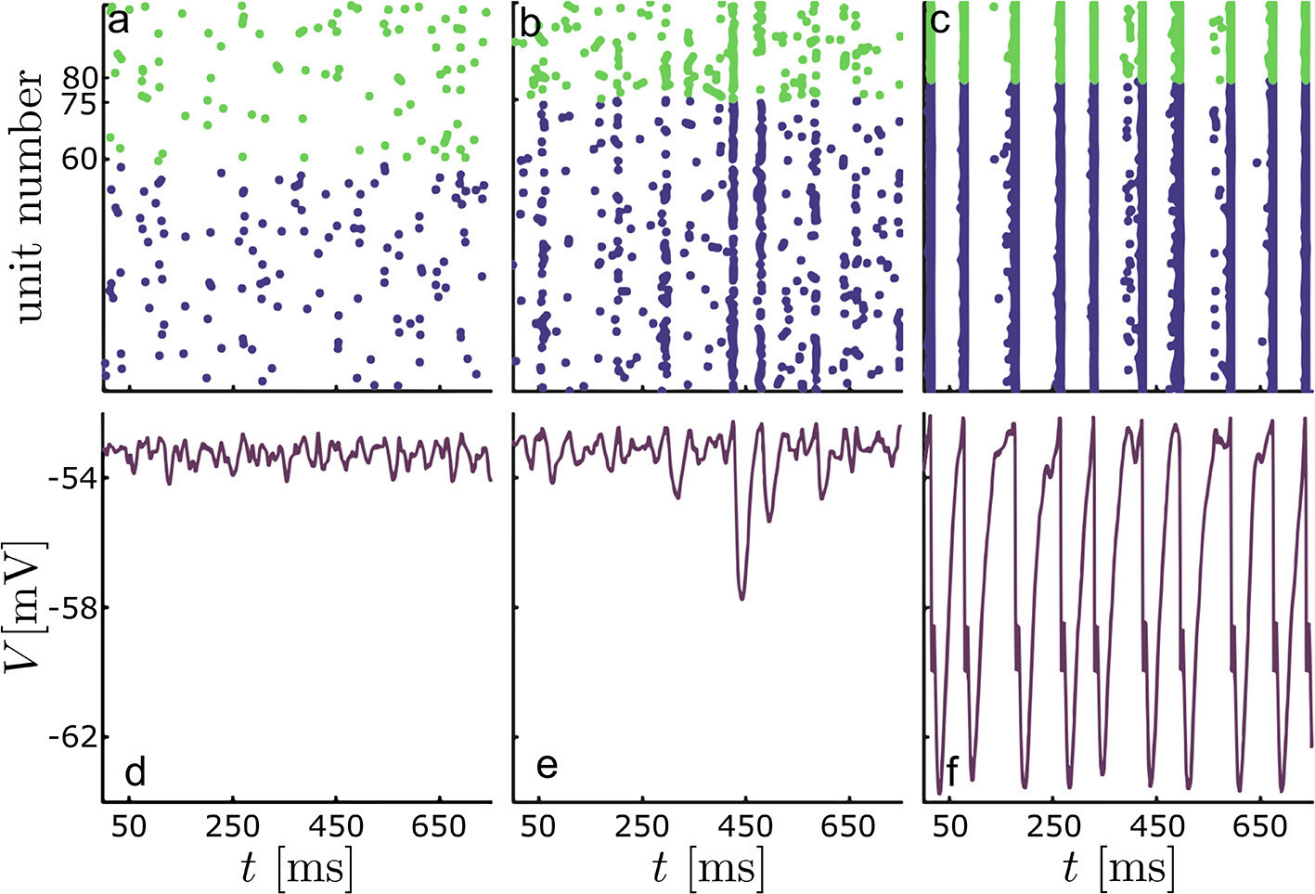
Typical behavior of the LIF network for different fractions of excitatory and inhibitory units. Top panels **(a-c)** contain raster plots for 10^4^ units, excitatory in blue and inhibitory in green. The bottom panels **(d-f)** show the corresponding LIF mean-field potential, *V*(*t*); **(a,d)** λ = 0.6, **(b,e)** λ = 0.75 and **(c,e)** λ = 0.8; in all cases the overall network degree was set to *p* = 0.2.

**Figure 2:**
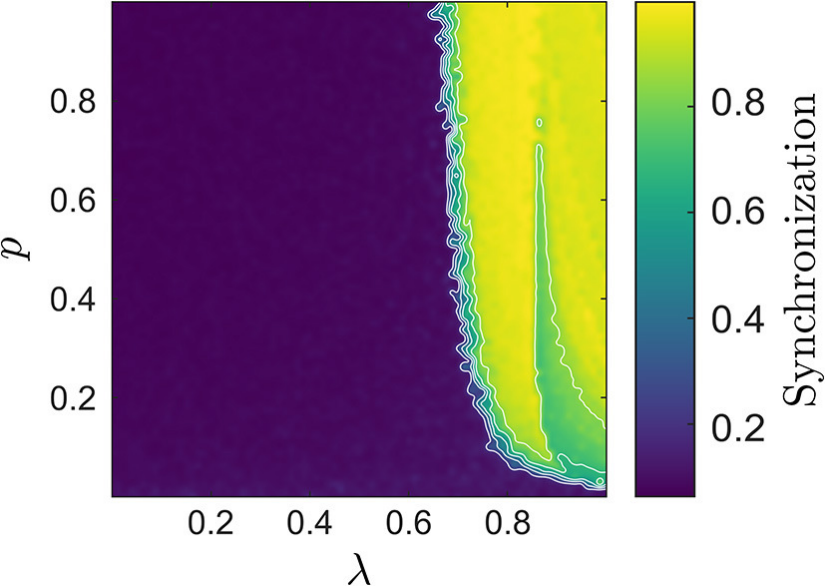
The synchronization degree in the {*p*, λ} space computed using the Spike-contrast measure (Ciba et al., 2018). A value close to 1 indicates strong synchronization, here particularly pronounced for large λ, whereas a value close to 0 indicates de-synchronized states. This is the case if *p* is very small, or if the number of inhibitory units exceeds that of the excitatory ones, i.e., if λ is small.

Before investigating the extent of agreement in more detail, we first verified that the chosen parameter range covered different dynamical regimes including phase transitions from de-synchronized to synchronized states. Figure 1 illustrates typical examples of the network with and without synchronization, while a more complete picture of the network's synchronization characteristics over the {*p*, λ} parameter space is given in Figure 2. There, we quantified the degree of synchrony using a spike train measure called *spike-contrast* (Ciba et al., 2018). In brief, one contrasts activity vs. non-activity (spike vs. no-spike) in temporal bins and varies the bin-size to obtain a time scale independent result. This allows for unraveling time scales of synchrony, over which we averaged here. Once the overall network degree exceeds a minimal value, increasing it further has little to no influence on the state of synchronization in the network. In contrast, altering λ, e.g., increasing the relative amount of excitatory units, one can observe a spontaneous switch between de-synchronized to synchronized states.

The degree of synchronization in the network plays a crucial role for the appropriateness of the neural mass models in relation to the average network dynamics. As said, there is particular interest in the spectral content of the dynamics of neural populations (Buzsaki, 2006; Başar, 2012). Hence, we first summarize our comparisons in the frequency domain.

### Spectral Characteristics

**Figure 3:**
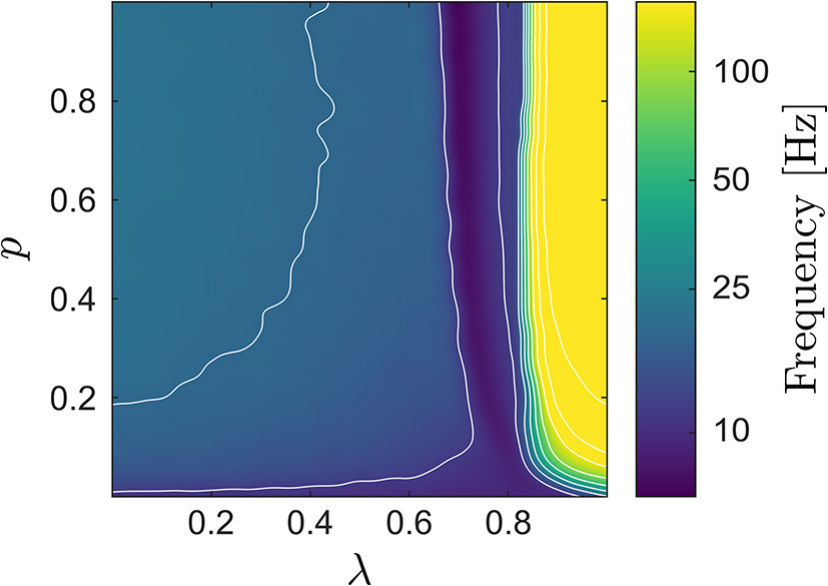
Median frequency of the LIF network's average potential *V*LIF; several contour lines were added to highlight the increase of the median frequency when increasing λ. When looking also at Figure 2, one can identify two transitions, one from the de-synchronized to a synchronized state (at low frequencies), followed by a second one from low- to high-frequency synchronization.

In what follows, we denote the average LIF network potential and the neural mass potentials by *V*LIF, *V*CFM, and *V*MFM, respectively, and refer to the corresponding spectra as *P*LIF, *P*CFM, and *P*MFM. Figure 3 shows the median frequency of *V*LIF, which in combination with Figure 2, provides a more encompassing view on the LIF network dynamics: when passing from small to larger values of λ, the network starts to synchronize. At the onset of synchrony the network's median frequency remains very low (this transition can be observed in Figure 1). When further increasing λ, this first transition from the de-synchronized to a synchronous state is followed by a second one, at which the network enters the synchronous regular regime with high spiking rate (Brunel and Hakim, 1999; Yger et al., 2011). The first transition appears smoother, which may be attributed to an effect of the chosen measure, i.e., the median frequency.

**Figure 4:**
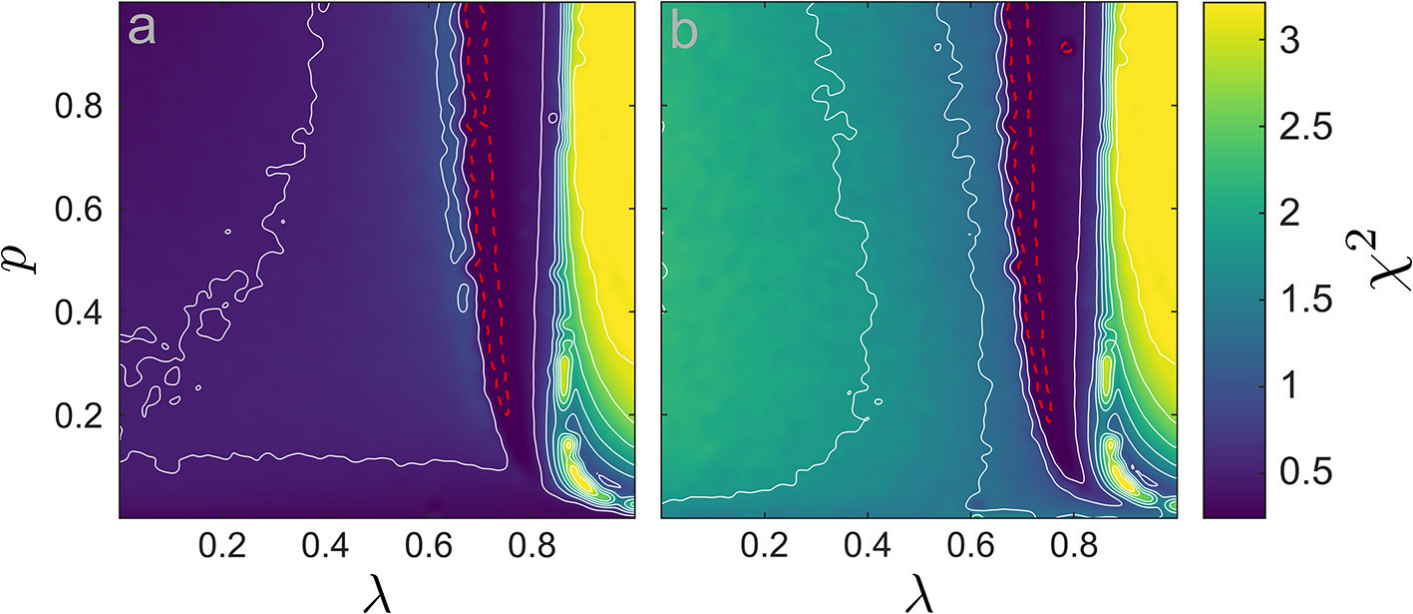
χ^2^-statistic computed between the power spectra (χ^2^(·, ·)) in the {*p*, λ} space (10^5^ values). **(a)** χ^2^ between the LIF network and the CFM, χ^2^(*P*LIF, *P*CFM). **(b)** χ^2^ between the LIF network and the MFM, χ^2^(*P*LIF, *P*MFM). We add several contour lines in white to improve legibility. The dashed-red line indicates the boundaries of significance region with α = 0.01 (conform the χ^2^ distribution): inside the small region encircle by the dashed-red line, the CFM/MFM spectra were not significantly different from the LIF network spectrum.

As expected, in the de-synchronized region both neural mass models displays significantly lower median frequencies than the network counterpart due to their low-pass filter characteristics. In all other dynamical regimes, the median frequencies seems to agree, at least at first glance. In general, one can expect that around a transition to or from synchrony, a spectral distribution may change qualitatively, rendering a comparison based solely on the median frequency incomplete. We therefore supplemented our analysis by a χ^2^-statistic (25) between the network's and the neural masses' power spectral densities shown in Figure 4 for both CFM and MFM; see the *Methods* section for details.

In the region of de-synchronization, the χ^2^-values between the LIF network and the MFM spectra are clearly larger than the CFM counterpart. That is, there, the CFM provides a better representation of the network's average potential; cf. Figure 4. In the synchronous network state both models generate similar χ^2^-values. In the region of regular high-frequency synchronization, both spectra substantially disagree with the *V*_LIF_ spectrum. There, the quality-of-fit is poor. By contrast, in the low-frequency synchronous region, especially close to the transition points from de-synchronization to synchronization, the spectra from both models agree to a level in which our χ^2^-statistics does not identify any statistically significant differences; see the areas encircled by the red dashed lines in Figure 4.

### Temporal Characteristics

**Figure 5:**
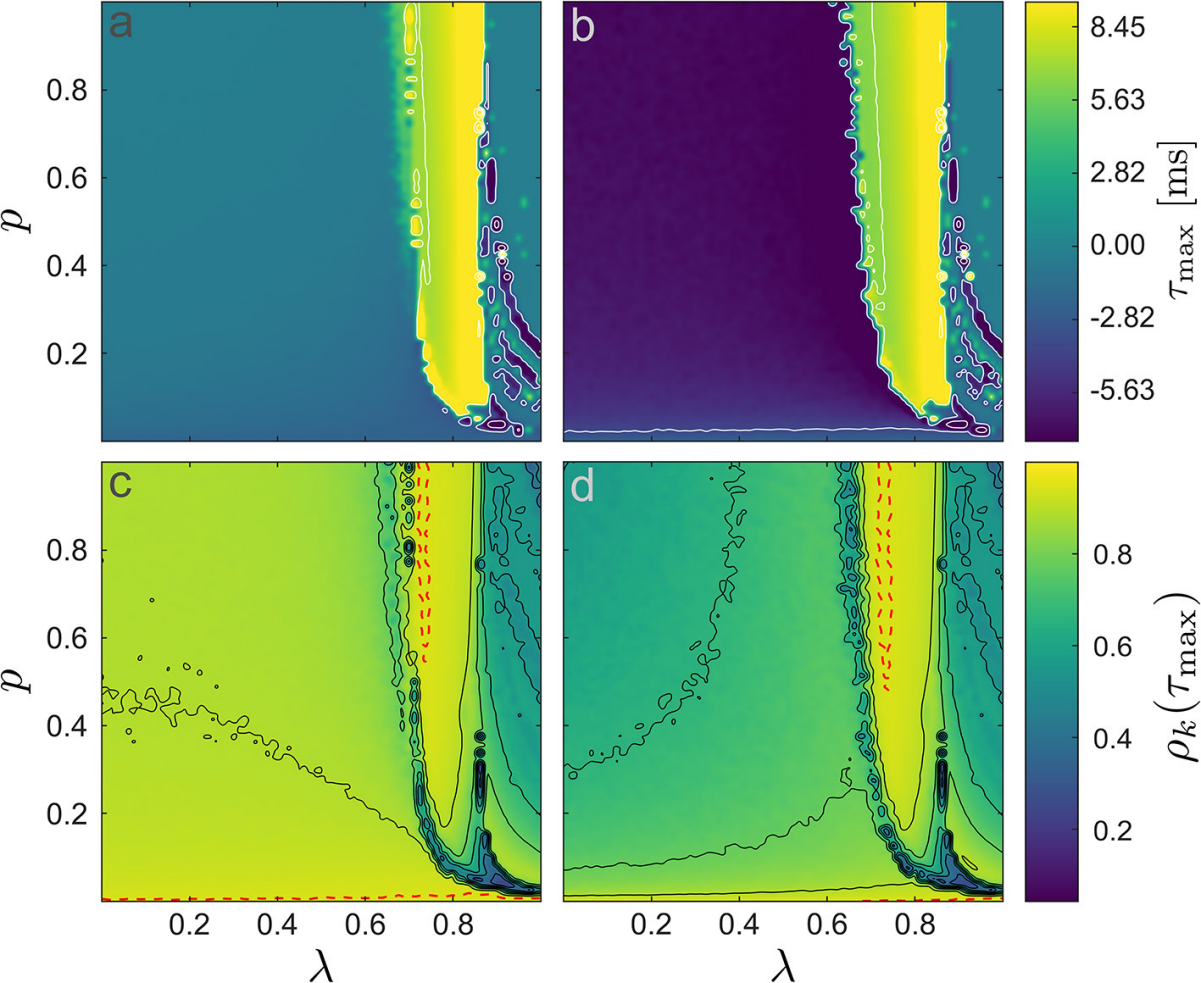
Time lags and correlation coefficients. **(a,b)** depict the optimal time lags τ_max_, **(a)** CFM and **(b)** MFM. We added contour lines (in white) to improve legibility. In **(b)** there is a change in the time lags when *p* is sufficiently large for the LIF network to generate spikes. In **(c,d)** the corresponding correlation coefficients ρ_*k*_(τ_max_) between the LIF model and **(c)** CFM and **(d)** MFM are shown. The red-dashed lines in panels **(c,d)** indicate boundaries of significance; α = 0.01 obtained by applying the Fisher transformation to the correlation values (Fisher, 1915). Inside the area defined by the red-dashed line in the synchronized region and the small area in the asynchronous region where *p* → 0, the time series of the two neural mass models were not significantly different than the LIF mean field.

We analyzed the *V*_CFM_ and *V*_MFM_ time series by determining the cross-correlation function between them and *V*_LIF_. In view of the aforementioned response characteristics, we expected the extrema of this correlation to be located at finite, non-vanishing time lags τ. Therefore, we first estimated these time lags and, subsequently, determined the corresponding correlation coefficient as ρ_*k*_(τ_max_) with

(4) $$\begin{array}{l} \rho_{k}\left(\tau \right)=\int {\hat{V}}_{\text{LIF}}\left(t\right){\hat{V}}_{k}\left(t+\tau \right)dt \end{array}$$

and $\tau_{\text{max}}=\arg\max_{\tau }|\rho_{k}\left(\tau \right)|$—in (4) the $\left(\hat{\cdot }\right)$ indicates the computation of z-scores and *k* ∈ {CFM, MFM}. The resulting time lags and maximum correlation values are shown in Figure 5. As expected from the close relationship between the neural mass model and the linear response, the time lags switch from zero to positive values around the points of transition between de-synchronized and synchronized states—the fact that in the de-synchronized regime, the MFM displays a negative time lag shows its deviation from the mere low-pass characteristics for CFM. The drop in time lag in the transition from low-frequency to high-frequency synchronization is unexpected and we return to it in the *Discussion* section below.

For both CFM and MFM, the correlation followed a {*p*, λ}-dependent pattern similar to that of the synchronization degree combined with the median frequency (Figures 2, 3). Apart from the regions with very small *p*, i.e., where the network was set to be very sparsely connected, the region with pronounced low-frequency synchronization (λ ∈ [0.70, 0.85]) is accompanied with the largest correlation values. Similar to the χ^2^-based approach for the spectra, we determined a significance interval for the ρ_*k*_(τ_max_), now after Fisher transform (Fisher, 1915). The red-dashed significance boundaries indicate regions of proper approximations. In line with the results for the power spectral densities, also here the agreement between neural mass models and the average LIF network dynamics becomes arbitrarily bad in the region of high-frequency synchronization (larger values of λ). While both models largely agreed there, they differ when the network is de-synchronized (λ < 0.7) where ρ*V*_CFM_ displayed larger correlation values than ρ_*V*MFM_.

## Discussion

We compared two neural mass models with the average potential of a network of LIF neurons. Both models provide limited approximations of the average network potential for large regions of the {*p*, λ} parameter space spanning network degree and the relation between excitatory and inhibitory neurons. We found arbitrary discrepancies between the neural mass models and the average potential of the LIF network, manifested in both the cross-correlation between model and network potentials and in the corresponding spectral densities that we assessed via χ^2^-statistics. These were not just minor quantitative deviations but qualitative and largely unpredictable ones. Only in a very confined region around the transition from the de-synchronized state to low-frequency synchronization in the LIF network, both models performed well. There, our simulations did not reveal any significant differences between the “real” average network potential and the outcome of the two neural mass dynamics. Although this finding is troublesome as it implies a quite limited accuracy of neural mass approximations—at least in some parameter regimes—, we want to emphasize that our models appear suitable for studying neural activity at the transition between synchronized and de-synchronous states. In fact, transition regimes have recently gained much interest as they seem to particularly characterize neural dynamics during resting state (Ito et al., 2005, 2007) and also may describe more general metastability in ongoing whole-brain activity (Tognoli and Kelso, 2014; Deco et al., 2017; Beim Graben et al., 2019).

We incorporated two neural mass models derived from microscopic conductance-based neural models. For the first one, we followed the procedure described by Rodrigues et al. (2010). As already mentioned in the *Introduction*, some parts of their derivation arguably lack some rigor. For small fluctuations of the driving forces, they assumed the latter to be constant across channels, i.e., the membrane potential was considered constant and identical for all neurons on the synaptic terms; see Equation (17). While Rodrigues et al. did mention that this may not be a valid assumption, they also claimed this approximation to be required for deriving their neural mass model. Being constant is a very strong constraint for the membrane potential, which has—to the best of our knowledge—not been supported by previous research. Moreover, to us remains unclear why this approximation has only been applied for the synaptic terms but nowhere else in Equation (16). For the second neural mass model, we conducted an alternative derivation with the same starting point (16). To compute the population mean, we separated the time scales of the neural dynamics. This led to a slightly more complicated dynamics and reducing it further required a second approximation: the expectation values of the spike trains have to be identical among the different units. While this may be true for homogeneous cases, it may not hold in general. Strikingly, however, our results for both CFM and MFM largely agree. Both represent fairly accurately the results of the LIF network mean-field around the onset of low-frequency synchronization, while in other regions of the {*p*, λ}-space they both performed arbitrarily bad. Given these poor performances, we hope for future work to focus on (even more) rigorous alternatives.

In the population modeled in this work, the heterogeneity has been included by a mixture of excitatory and inhibitory units, while the external and internal connectivities are uniformly distributed and the different type of units are identical among them. This simplified the implementation of the models and the interpretation of our results. Yet, we have to admit that —when it comes to biological plausibility—this choice might be considered unrealistic: the homogeneity between neurons of the same type can be challenged (Reyes et al., 1998; Jinno et al., 2007; Ávila-Åkerberg et al., 2010) and the uniform distribution of connectivity might be replaced by, e.g., small-world topologies (Bettencourt et al., 2007; van den Heuvel et al., 2016; Bassett and Bullmore, 2017). Here we would like to add that using the current modeling approach the cell-to-cell heterogeneity including their role in neural coding has been explored elsewhere (Boustani and Destexhe, 2009; Mejias and Longtin, 2012, 2014; Carlu et al., 2020) while the modeling of small-world, modular and more realistic topologies remains future work.

As a final remark we would like to point out that discrepancies between microscopic and macroscopic descriptions for the same neural network are problematic when seeking for inferences from one level to the other. However, this ability for such inferences is fundamental, since a model has only value when it allows for predictions. Studying the network on the macroscopic neural mass level should allow to forecast a dynamics that could be verified on the microscopic, i.e., full network level. Of course, this requires a proper modeling of the latter. Since this cannot be guaranteed under all circumstances, the litmus test remains to forecast experimental data. This, however, will come with further challenges as one has to answer, e.g., “what is the microscopic level?” or “what defines the *full* network?”. For instance, in encephalography the likelihood that the recorded potentials of some cortical region contains large contributions of (tangentially oriented) inhibitory units is arguably small. That is, although the full network does contain inhibitory units the recorded mean values of the underlying neural population may not cover them. In the Appendix we sought to mimic this case by repeating our comparisons after selecting only excitatory units from the simulated LIF-network. Also in this case our results stay intact rendering our conclusions valid and possibly transferrable to this type of experimental data.

## Conclusion

Neural masses are common tools to model neural population dynamics. They are believed to mimic selected brain activity patterns with great accuracy. We questioned the relation between these models and the underlying spiking neural network. For populations with both excitatory and inhibitory neurons and random connectivity, we found that approximations via the corresponding mean-field dynamics may deviate arbitrarily from the network's average potential. Deviations may be particularly large when the network is either de-synchronized or fully synchronized and spikes at high rate, while mass models can fit well around the onset of low-frequency synchronization. Neural mass models covering several dynamical regimes require more than mere mean-field approximations because they typically average out the (synchrony-defining) spiking behavior.

## Methods

We will derive two neural mass models from a network of spiking neurons and compare them against the mean outcome of that network. The first model represents the CFM, and the second one contains a (slight) modification by means of a weakly non-linear response, i.e., the MFM. Then, our approach to test the model is the following: We simulate the spiking network for different values of two major topological parameters, i.e., the fraction of excitatory/inhibitory units and mean degree of the (random) network quantifying the general connectivity. We choose the parameters such that the dynamics undergoes a phase transition from the de-synchronized to a synchronized state (Yger et al., 2011). Throughout the simulations, we “record” both the output spiking activity and the mean membrane potential. While the latter is considered as reference, i.e., the “real” mean network activity, the first serves as input to the two neural mass models. Finally, we compare the outcome of the neural mass models with the real mean network activity in both the time and the frequency domain.

Below, we will specify the microscopic neuron and synapse dynamics and put them on a homogeneous network before deriving the two versions of the macroscopic Freeman model. Finally, we will provide all details about how we altered the network structure when probing model validity.

### Microscopic Dynamics

We consider a population or network of *n* = 1, …, *N* neurons where neuron *n* is described in terms of the dynamics of its membrane potential *v*_*n*_ = *v*_*n*_(*t*) and voltage- and time-dependent conductances. If *c*_*n*_ and *g*_*n*_ denote the membrane's capacitance and leak conductance, respectively, then the dynamics can be cast in the form

(5) $$\begin{array}{l} \tau_{n}dv_{n}+f_{n}\left(v_{n}\right)dt=g_{n}^{-1}j_{n}\left(t\right)dt+dw_{n}. \end{array}$$

The function *f*_*n*_(·) is—as of yet—generic and describes the voltage-dependent decay, *j*_*n*_(*t*) is the total current applied to neuron *n*. The membrane's time constant τ^(mem)^ can be given by its capacitance and leak conductance in terms of τ_*n*_: = *c*_*n*_/*g*_*n*_. And, *w* denotes a stochastic force summarizing random voltage fluctuations of the membrane; here, *w* will always reflect zero-centered, δ-correlated (white) Gaussian noise with variance *Q*. In what follows, we will specify both *f*_*n*_(·) and *j*_*n*_(*t*) and estimate the expectation values of the population average for finite *N*.

We first notice that the input current *j*_*n*_(*t*) can be a combination of an internal current generated within the network and an external one stemming from outside the network. We denote them as $j_{n}^{\left(\text{net}\right)}$and $j_{n}^{\left(\text{ext}\right)}$, respectively, and assume that they superimpose like $j_{n}=j_{n}^{\left(\text{net}\right)}+j_{n}^{\left(\text{ext}\right)}$. Without loss of generality, the internal current will be given as

(6a) $$\begin{array}{l} j_{n}^{\left(\text{net}\right)}=-\underset{\sigma \in \left\{E,I\right\}}{\sum }\overset{N}{\underset{m=1}{\sum }}g_{nm}^{\left(\sigma \right)}\left(v_{n}-{ṽ}_{nm}^{\left(\sigma \right)}\right) \end{array}$$

where ${ṽ}_{nm}^{\left(\sigma \right)}$ is the reversal potential for a synapse between neurons *n* and *m*. The synapse can be excitatory or inhibitory, which we indicate by σ = *E* or σ = *I*, respectively. The synaptic activity is further quantified by a time-dependent conductance $g_{nm}^{\left(\sigma \right)}$ that depends on incoming spikes. We consider the corresponding response to be cast into a first-order, linear dynamics, i.e., we include so-called exponential synapses with conductance dynamics which leads to the dynamics

(6b) $$\begin{array}{l} \tau_{nm}^{\left(\sigma \right)}dg_{nm}^{\left(\sigma \right)}=-\left(g_{nm}^{\left(\sigma \right)}-{ĝ}_{nm}^{\left(\sigma \right)}\phi_{nm}^{\left(\sigma \right)}\right)dt. \end{array}$$

The parameter ${ĝ}_{nm}^{\left(\sigma \right)}$ relates to the maximum conductance, $\tau_{nm}^{\left(\sigma \right)}$ is the characteristic time of the type-σ synapse between neurons *n* and *m*, and $\phi_{nm}^{\left(\sigma \right)}$ is the input that neuron *n* receives from neuron *m*. If that input is composed of spikes, it can be cast into the form

(6c) $$\begin{array}{l} \phi_{nm}^{\left(\sigma \right)}=A_{nm}\underset{k}{\sum }\delta \left(t-t_{m,k}^{\left(\sigma \right)}\right) \end{array}$$

where *A*_*nm*_ denotes the elements of the network's adjacency matrix, i.e., *A*_*nm*_ = 1 if neuron *m* targets neuron *n* and 0 otherwise, and $\sum_{k}\delta \left(t-t_{m,k}^{\left(\sigma \right)}\right)$ is a spike train emitted by neuron *m* with spikes at times $t_{m,k}^{\left(\sigma \right)}$. Similarly, the external current may be expressed as

(7a) $$\begin{array}{l} j_{n}^{\left(\text{ext}\right)}=-\overset{M}{\underset{m=1}{\sum }}g_{nm}^{\left(\text{ext}\right)}\left(v_{n}-{ṽ}_{nm}^{\left(\text{ext}\right)}\right) \end{array}$$

given *M* external units that project into the network with external synaptic conductivity and external inputs of the form

(7b) $$\begin{array}{l} \tau_{nm}^{\left(\text{ext}\right)}dg_{nm}^{\left(\text{ext}\right)}=-\left(g_{nm}^{\left(\text{ext}\right)}-{ĝ}_{nm}^{\left(\text{ext}\right)}\phi_{nm}^{\left(\text{ext}\right)}\right)dt \end{array}$$

and

(7c) $$\begin{array}{l} \phi_{nm}^{\left(\text{ext}\right)}=B_{nm}\underset{k}{\sum }\delta \left(t-t_{m,k}^{\left(\text{ext}\right)}\right) \end{array}$$

respectively. The parameter ${ĝ}_{nm}^{\left(\text{ext}\right)}$ is again related to the maximum conductance, $\tau_{nm}^{\left(\text{ext}\right)}$ denotes the characteristic time of the corresponding synapse and $\phi_{nm}^{\left(\text{ext}\right)}$ is an external spike train that enters according to the adjacency matrix between the external and internal neurons (*B*_*nm*_ = 1 if the external neuron *m* targets internal neuron *n* and 0 otherwise).

We would like to note that, thus far, we did not detail the dynamics of the individual neuron *n*, i.e., the function *f*(·) can still be arbitrary (except that it has to be integrable). Put differently, the system (5) and (6a-c) covers a very general case for a conductance-based, stochastic spiking network model under impact of an external drive (7a-c).

Next, in order to make this system tractable, we consider the case in which all synapses of type σ are identical for every neuron. This means that

(8) $$\begin{array}{l} \forall_{m=1,\ldots ,M}:\;\left\{\begin{array}{cc} \tau_{nm}^{\left(\sigma \right)}=: & \tau_{n}^{\left(\sigma \right)}\\ {ĝ}_{nm}^{\left(\sigma \right)}=: & {ĝ}_{n}^{\left(\sigma \right)}\\ {ṽ}_{nm}^{\left(\sigma \right)}=: & {ṽ}_{n}^{\left(\sigma \right)} \end{array}\right\}, \end{array}$$

i.e., all synapses have identical characteristic times, maximum conductances, and reversal potentials. For the sake of legibility, we further introduce two abbreviations, namely

(9) $$\begin{array}{l} g_{n}^{\left(\sigma \right)}:=\overset{N}{\underset{m=1}{\sum }}g_{nm}^{\left(\sigma \right)}\text{ and }\phi_{n}^{\left(\sigma \right)}:=\overset{N}{\underset{m=1}{\sum }}\phi_{nm}^{\left(\sigma \right)} \end{array}$$

which represent the total conductivity of type-σ synapses in neuron *n* and the total spike input via type-σ synapses received by neuron *n*, respectively. Then, substituting (8) and computing the sum over *m* in (6b) yields

(10) $$\begin{array}{l} \tau_{n}^{\left(\sigma \right)}dg_{n}^{\left(\sigma \right)}=-\left(g_{n}^{\left(\sigma \right)}-{ĝ}_{n}^{\left(\sigma \right)}\phi_{n}^{\left(\sigma \right)}\right)dt. \end{array}$$

### Network of LIF Neurons

The arguably simplest case of spiking neurons are LIF neurons. To model them, we constrain *f*_*n*_(·) to be linear in *v*_*n*_. In more detail, we define *f*_*n*_(*v*_*n*_) = (*v*_*n*_ − ṽ_*n*_), where ṽ_*n*_ denotes the membrane reversal potential. We add a further homogeneity assumptions by considering identical *f*_*n*_ as well as identical membrane characteristics for all neurons, i.e., *c*_*n*_ =:*c*, $g_{n}=:g\Rightarrow \tau_{n}=:\tau^{\left(\text{mem}\right)}$, and ṽ_*n*_ =:ṽ. Likewise, we assume homogeneity of the synapse by setting $\tau_{n}^{\left(\sigma \right)}=:6\tau^{\left(\sigma \right)}$, ${ṽ}_{n}^{\left(\sigma \right)}=:{ṽ}^{\left(\sigma \right)}$, and ${ĝ}_{n}^{\left(\sigma \right)}=:{ĝ}^{\left(\sigma \right)}$, i.e., all synapses of the same type σ are identical across the population^2^. Using (10) and the homogeneity, we can simplify the system (5) and (6) as

(11a) $$\begin{array}{l} \tau^{\left(\text{mem}\right)}dv_{n}=-[\left(v_{n}-\tilde{v}\right)+\\ \;+\frac{1}{g}\;\sum_{\sigma \in \{E,I\}}\;\;g_{n}^{\left(\sigma \right)}\;\left(v_{n}-{\tilde{v}}^{\left(\sigma \right)}\right)]dt+dw_{n} \end{array}$$

and

(11b) $$\begin{array}{l} \tau^{\left(\sigma \right)}dg_{n}^{\left(\sigma \right)}=-\left(g_{n}^{\left(\sigma \right)}-{ĝ}^{\left(\sigma \right)}\phi_{n}^{\left(\sigma \right)}\right)dt \end{array}$$

with

(11c) $$\begin{array}{l} \phi_{n}^{\left(\sigma \right)}=\overset{N}{\underset{m=1}{\sum }}A_{nm}\sum_{k}\delta \left(t-t_{m,k}^{\left(\sigma \right)}\right). \end{array}$$

Finally, the membrane dynamics is supplemented by the reset rule that reads

(11d) $$\begin{array}{l} if\;v_{n}\;reaches\;v^{\left(\text{thres}\right)},\;then\;neuron\;n\;emits\;a\;spike\\ \text{ -}its\;membrane\;potential\;v_{n}\;resets\;to\;v^{\left(\text{reset}\right)}\\ \text{ -}and\;stays\;there\;for\;a\;refractory\;period\;\tau^{\left(\text{ref}\right)}. \end{array}$$

The set of equations (11a-d) defines our microscopic dynamics. This dynamics can be readily completed by adding external input as defined in (7a-c) much in line with the formulation of (11b) and (11c).

### Macroscopic Dynamics

In the following we will estimate the population mean of the membrane potential's expectation value—recall that the dynamics (11a) contains noise that we “eliminate” by determining first the dynamics' first moment *V*_*n*_: = 〈*v*_*n*_〉. Hence, the task is to approximate

(12) $$\begin{array}{l} V:=\frac{1}{N}\overset{N}{\underset{n=1}{\sum }}V_{n}:=\frac{1}{N}\overset{N}{\underset{n=1}{\sum }}\langle v_{n}\rangle . \end{array}$$

Before doing so, however, we recast (11b) in the form

(13) $$\begin{array}{l} \left[\tau^{\left(\sigma \right)}\frac{d}{dt}+1\right]\langle g_{n}^{\left(\sigma \right)}\rangle ={ĝ}^{\left(\sigma \right)}\Phi_{n}^{\left(\sigma \right)} \end{array}$$

where we introduced the first moment of the spike trains, i.e.,

(14) $$\begin{array}{l} \Phi_{n}^{\left(\sigma \right)}:=\langle \phi_{n}^{\left(\sigma \right)}\rangle . \end{array}$$

By construction, 〈*w*_*n*_〉 = 0 holds, with which we find

(15) $$\begin{array}{l} \left[\tau^{\left(\text{mem}\right)}\frac{d}{dt}+1\right]V=\\ \;ṽ-\frac{1}{g}\underset{\sigma \in \left\{E,I\right\}}{\sum }\frac{1}{N}\overset{N}{\underset{n=1}{\sum }}\left(\langle g_{n}^{\left(\sigma \right)}v_{n}\rangle -\langle g_{n}^{\left(\sigma \right)}\rangle {ṽ}^{\left(\sigma \right)}\right). \end{array}$$

We can combine (13) and (15), in particular, when assuming identical time constants across synapse types σ, i.e., ∀ σ:τ^(σ)^ =* :τ*^(syn)^. Then, we find

(16) $$\begin{array}{l} \left[\tau^{\left(\text{mem}\right)}\frac{d}{dt}+1\right]\left[\tau^{\left(\text{syn}\right)}\frac{d}{dt}+1\right]V=\\ \tilde{v}+\sum_{\sigma \in \{E,I\}}\frac{{\hat{g}}^{\left(\sigma \right)}}{g}{\tilde{v}}^{\left(\sigma \right)}\Phi^{\left(\sigma \right)}-\frac{1}{g}\sum_{\sigma \in \{E,I\}}\frac{1}{N}\sum_{n=1}^{N}\left[\tau^{\left(\text{syn}\right)}\frac{d}{dt}+1\right]\langle g_{n}^{\left(\sigma \right)}v_{n}\rangle \end{array}$$

with $\Phi^{\left(\sigma \right)}:=N^{-1}\overset{N}{\underset{n=1}{\sum }}\Phi_{n}^{\left(\sigma \right)}$. The last term on the right-hand side of (16) needs to be approximated, and the way of which discriminates our two models. We first adopt the line of reasoning by Rodrigues et al. (2010) leading to the CFM before presenting a slight adjustment culminating in the MFM (cf. Tewarie, 2014, chap. 2).

#### The Conventional Freeman Model (CFM)

Approximating the term $\langle g_{n}^{\left(\sigma \right)}v_{n}\rangle$ in (15) can be difficult because smallness arguments may not hold in view of the stochastic nature of the dynamics. Rodrigues et al. (2010) introduced an admittedly gross step by considering

(17) $$\begin{array}{l} \langle g_{n}^{\left(\sigma \right)}v_{n}\rangle \approx \langle g_{n}^{\left(\sigma \right)}\overset{-}{V}\rangle =\langle g_{n}^{\left(\sigma \right)}\rangle \overset{-}{V} \end{array}$$

where $\overset{-}{V}$ denotes the constant mean membrane potential of the population. This approximation implies that the individual membrane potentials *v*_*n*_ are arbitrarily close to the population mean $\overset{-}{V}$, averaged over time. Note that when applying this approximation one selectively ignores all of their dynamic characteristics on the right-hand side of (16); cf. *Discussion* section (but not on the left-hand side). Presuming this is acceptable, the last term on the right-hand side of (16) simplifies drastically because of

(18) $$\begin{array}{l} \left[\tau^{\left(\text{syn}\right)}\frac{d}{dt}+1\right]\langle g_{n}^{\left(\sigma \right)}v_{n}\rangle \approx \\ \;\left[\tau^{\left(\text{syn}\right)}\frac{d}{dt}+1\right]\langle g_{n}^{\left(\sigma \right)}\rangle \overset{-}{V}\overset{\left(13\right)}{=}{ĝ}^{\left(\sigma \right)}\hat{V}\Phi_{n}^{\left(\sigma \right)} \end{array}$$

Substituting (18) into (16) yields

(19) $$\begin{array}{l} \left[\tau^{\left(\text{mem}\right)}\frac{d}{dt}+1\right]\left[\tau^{\left(\text{syn}\right)}\frac{d}{dt}+1\right]V=\\ \;ṽ-\underset{\sigma \in \left\{E,I\right\}}{\sum }\frac{{ĝ}^{\left(\sigma \right)}}{g}\left(\overset{-}{V}-{ṽ}^{\left(\sigma \right)}\right)\Phi^{\left(\sigma \right)}. \end{array}$$

In the presence of external input, as given in (7a-c), the full dynamics finally reads

(20) $$\begin{array}{l} \left[\tau^{\left(\text{mem}\right)}\frac{d}{dt}+1\right]\left[\tau^{\left(\text{syn}\right)}\frac{d}{dt}+1\right]V=ṽ-\underset{\sigma \in \left\{E,I\right\}}{\sum }\frac{{ĝ}^{\left(\sigma \right)}}{g}\left(\overset{-}{V}-{ṽ}^{\left(\sigma \right)}\right)\Phi^{\left(\sigma \right)}\\ \;-\frac{{ĝ}^{\left(\text{ext}\right)}}{g}\left(\overset{-}{V}-{ṽ}^{\left(\text{ext}\right)}\right)\Phi^{\left(\text{ext}\right)}. \end{array}$$

In our study, the function Φ^(ext)^ consists of Poisson spike trains as specified in Equation (7c).

Both forms, (19) and (20), agree entirely with the Freeman model (1) when identifying α = 1/τ^(mem)^, β = 1/τ^(syn)^, and *J* = rhs(19) or *J* = rhs(20).

#### The Modified Freeman Model (MFM)

For an alternative approximation of the term $\langle g_{n}^{\left(\sigma \right)}v_{n}\rangle$ in (16), let us detail the time scales, at which the membrane potentials and the synapses evolve. Synaptic time constants can be as small as 1.7 ms (Häusser and Roth, 1997), much in the range of typical time scales of the membrane dynamics. Yet, changes in most chemical synapses are much slower than the changes the membrane potential, in particular, the generation/emission of action potentials. Then, one may assume that the membrane potential instantly follows changes at the synapse, its dynamics can be eliminated adiabatically, i.e., we can use

(21) $$\begin{array}{l} \left|\frac{dv_{n}}{dt}/v_{n}\right|\;\ll \;\left|\;\frac{dg_{n}^{\left(\sigma \right)}}{dt}/g_{n}^{\left(\sigma \right)}\;\right| \end{array}$$

to rewrite

(22) $$\begin{array}{l} \left[\tau^{\left(\text{syn}\right)}\frac{d}{dt}+1\right]\langle g_{n}^{\left(\sigma \right)}v_{n}\rangle \approx \\ \langle \left(\left[\tau^{\left(\text{syn}\right)}\frac{d}{dt}+1\right]g_{n}^{\left(\sigma \right)}\right)v_{n}\rangle \overset{\left(13\right)}{=}{ĝ}^{\left(\sigma \right)}\Phi_{n}^{\left(\sigma \right)}\langle v_{n}\rangle . \end{array}$$

While this approximation contains sufficient rigor under the proviso of a proper time scale separation, we also require that

(23) $$\begin{array}{l} \frac{1}{N}\overset{N}{\underset{n=1}{\sum }}{ĝ}^{\left(\sigma \right)}\Phi_{n}^{\left(\sigma \right)}\langle v_{n}\rangle \approx {ĝ}^{\left(\sigma \right)}\Phi^{\left(\sigma \right)}V \end{array}$$

which is true for Φ being the external spike train but may be arbitrarily inaccurate for the internal one Φ^(σ)^—again we refer to the *Discussion* section for a critical review. If this approximation turns out adequate, the MFM becomes

(24) $$\begin{array}{l} \left[\tau^{\left(\text{mem}\right)}\frac{d}{dt}+1\right]\left[\tau^{\left(\text{syn}\right)}\frac{d}{dt}+1\right]V=\\ ṽ-\underset{\sigma \in \left\{E,I\right\}}{\sum }\frac{{ĝ}^{\left(\sigma \right)}}{g}\left(V-{ṽ}^{\left(\sigma \right)}\right)\Phi^{\left(\sigma \right)}-\frac{{ĝ}^{\left(\text{ext}\right)}}{g}\left(V-{ṽ}^{\left(\text{ext}\right)}\right)\Phi^{\left(\text{ext}\right)}. \end{array}$$

In contrast to (20), the dynamics (24) contains a parametric forcing since on the right-hand side the constant $\overset{-}{V}$ is replaced by the time-dependent mean potential *V*. Note, however, that despite this difference our simulation results revealed that given the chosen parameter values (õver-damped second-order response) the outcome of both models (20) and (24) largely agree.

### Numerical Methods

#### Simulations

We simulated *N* = 10, 000 LIF neurons with three types of synapses, each. The network equations were integrated using an Euler-Maruyama scheme with a time step of Δ*t* = 0.1 ms and noise variance *Q* = 5·10^−4^ for a total duration of *T* = 3·10^4^ ms, i.e., for 3·10^5^ time steps. We discarded a transient regime of $T_{0}=3\cdot 10^{3}$ ms. The network was stimulated by 10, 000 independent Poisson trains each of them connected to each neuron in the network with probability *p*^(*ext*)^. The population average of the total spike input of each synaptic type σ received by each neuron at each time step *t*, ϕ^(σ)^ was stored as it subsequently served as input to the Freeman model. The temporal average of the population mean, $1/\left(T-T_{0}\right)\int_{T_{0}}^{T}Vdt$ served as proxy of $\overset{-}{V}$. For the neural masses, we employed a simple Euler forward scheme with the same time parameters used for the network model. The time constant τ^(syn)^ was set to 5 ms, i.e., the average of the synaptic time constants in the network; see Table 1.

**Figure 6:**
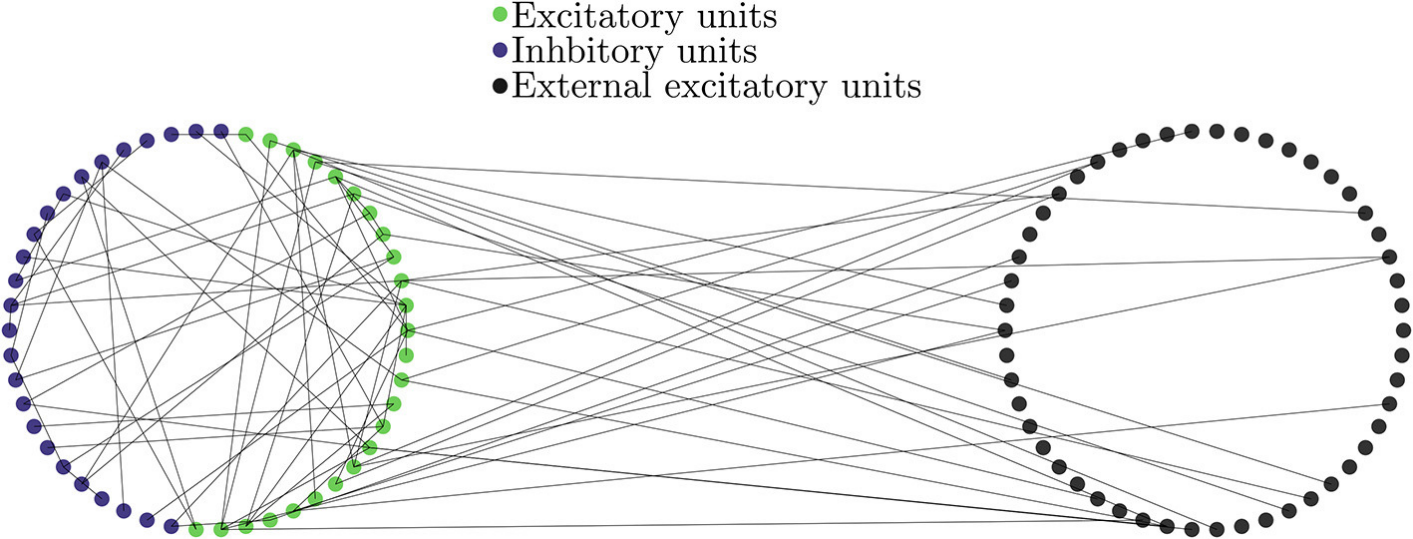
Diagram of the network of LIF units **(Left)** together with the external de-correlated input **(Right)**. Blue denotes excitatory, green inhibitory and black excitatory external neurons. The units on the LIF network are connected to each other with probability *p* independently on their type. The external units are modeled as independent Poisson trains and are connected with the same probability *p*^(*ext*)^ only to excitatory LIF units and are not connected to inhibitory ones.

To generate external input as Poisson spike trains, we drew random numbers from an exponential distribution. Since we drew the numbers at every time step for all the *M* = 10, 000 external units, we minimized the computational load by following (Zenke and Gerstner, 2014) and used that the union of distinct exponential distributions is again exponential. The mean frequency ν^(ext)^ of the external input was set to 5 Hz. The Erdős-Rényi adjacency {*A*_*nm*_} was constructed using the Gilbert model published in Batagelj and Brandes (2005), adjusted for directed graphs. For the connection probability, we used a range of *p* = 0…1 implying a range of mean degrees of *k* = *Np* = 0…*N*. The distribution of excitatory vis-à-vis inhibitory neurons was quantified by the ratio given by (3), i.e.,

$$\lambda :=\frac{\#excitatory}{\#inhibitory+\#excitatory}$$

with *#inhibitory* + *#excitatory* = *N* = 10, 000. This network structure is similar to that in Brunel and Wang (2003) and Mazzoni et al. (2015) and has been considered as a good estimator of cortical activity (Mazzoni et al., 2015). Note, however, that it differs from other LIF networks such as the ones used in Brunel (2000) and Wong and Wang (2006) in their external drive: in the current work only excitatory neurons receive external input. The internal network connectivity is given by directed Erdős-Rényi network without discriminating excitatory and inhibitory units. The connectivity between the external Poisson trains and the network of LIF neurons was also given by a directed Erdős-Rényi network with mean out degree (*Mp*^(ext)^); cf. Figure 6.

#### Parameter Values

The major parameters are summarized in Table 1. They largely agree with the settings in Yger et al. (2011) and resemble bio-physically plausible values.

#### Data Analysis

Per point {*p*, λ} in the parameter space, the network was simulated. We first verified that the chosen parameter range in fact covered the regime at which phase transitions from the de-synchronized to a synchronized state may occur by using a recently introduced, time-scale independent spike train synchrony measure coined *Spike-contrast* (Ciba et al., 2018). This measure yields results that are comparable to those of the well-established *Spike-distance* (Kreuz et al., 2013) but had our preference for its computational efficiency, which was necessary for our fairly large number of neurons. Subsequently, the regenerated internal and external spike trains served as input to the Freeman model. From the time series of the network's mean membrane potential and of the Freeman model's outcome we estimate power spectra via a discrete Fourier transform after boundary correction using a Hamming window. This procedure was repeated 10 times yielding average discrete power spectra *P*_ω_ as sample mean approximation of the power spectral densities. The corresponding median frequency ϖ served as first, albeit very gross outcome measure to compare the spectra of the original network (i.e., its average potential) vis-á-vis the spectra of our models, CFM and MFM.

To quantify the agreement between spectra, we used a χ^2^-statistics: Given two discrete spectra *P* = (*P*_1_, *P*_2_, …, *P*_*L*_) and *Q* = (*Q*_1_, *Q*_2_, …, *Q*_*L*_), their χ^2^-statistic can be given as

(25) $$\begin{array}{l} \chi^{2}\left(P,Q\right)=\overset{L}{\underset{l=1}{\sum }}\frac{{\left(P_{l}-Q_{l}\right)}^{2}}{P_{l}+Q_{l}} \end{array}$$

where the sum covers all *L* frequency components of the spectra (Press et al., 1989). Prior to using (25), the spectra were normalized to resemble histograms rather than probabilities.

## Data Availability Statement

The raw data supporting the conclusions of this article will be made available by the authors, without undue reservation.

## Author Contributions

ND and AD conceived and designed the study. AD derived the neural mass models. ND conducted the numerical simulations and all the data analysis. The first draft of the manuscript was written by ND and all authors commented on previous versions of the manuscript. All authors read and approved the final manuscript.

## Conflict of Interest

The authors declare that the research was conducted in the absence of any commercial or financial relationships that could be construed as a potential conflict of interest.

## Footnotes

^1^Ideally one can consider the thermodynamic limit, i.e., infinitely many neurons, though symmetries may allow for exact mean field description even for finite population sizes. Yet, the Freeman model is often considered appropriate for describing the “coordinated activity of cell assemblies of ~10^4^ neurons with ~10^8^ synapses”, Walter J. Freeman and Harry Erwin (2008), *Scholarpedia*, 3(2):3238.

^2^This appears a reasonable assumption given that the variability between neurons of the same type might be lost in the presence of noise.
