# Supplementary material for: On the Validity of Neural Mass Models
Source: Front Comput Neurosci. 2021 Jan 5;14:581040. doi: 10.3389/fncom.2020.581040 (PMC7814001; doi:10.3389/fncom.2020.581040)
Supplement: Supplementary file 1 [file Table_1.pdf]

On the Validity of Neural Mass Models: Appendix

Nicolás Deschle,[1,2] Juan Ignacio Gossn,[3] Prejaas Tewarie,[4,5] Björn Schelter,[2] and Andreas Daffertshofer[1]

[1]*Faculty of Behavioural and Movement Sciences, Amsterdam Movement Sciences & Institute for Brain and Behavior Amsterdam, Vrije Universiteit Amsterdam, van der Boechorststraat 9, Amsterdam 1081 BT, The Netherlands*

[2]*Institute for Complex Systems and Mathematical Biology, University of Aberdeen, King's College, Old Aberdeen AB24 3UE, United Kingdom*

[3]*Instituto de Astronomía y Física del Espacio, Consejo Nacional de Investigaciones Científicas y Técnicas, Universidad de Buenos Aires, Ciudad Universitaria, Ciudad de Buenos Aires, C1428ZAA, Argentina*

[4]*Sir Peter Mansfield Imaging Centre, School of Physics and Astronomy, University of Nottingham, Nottingham, United Kingdom*

[5]*Department of Clinical Neurophysiology and MEG Center, Amsterdam UMC, Vrije Universiteit Amsterdam, Amsterdam Neuroscience, Amsterdam, The Netherlands*

(Dated: December 12, 2020)

Frequency response function

Being a linear time-invariant system, the frequency response function can the computed for the CFM. Furthermore, as we show in the following, for the case of a fully connected network ($p = 1$), the CFM can be exactly expressed as a low-pass filter of the LIF mean-field. Given the Fourier transforms of the potential field $v = \mathcal{F}[V](\omega)$ and the input $j = \mathcal{F}[J](\omega)$, the Fourier transform of (1) reads

$$\left[-(2\pi\omega)^2 + 2\pi i(\alpha+\beta)\omega + \alpha\beta\right] v = \alpha\beta j \ , \tag{A.1}$$

where $i = \sqrt{-1}$ stands for the imaginary unit. Taking the square of the modulo in (A.1) yields

$$P_V = G \cdot P_J \tag{A.2}$$

where $P_V = |v|^2$ and $P_J = |j|^2$ are the power spectra of the potential V and the input J, respectively, and

$$G = \frac{\alpha^2\beta^2}{\left[\alpha^2 + (2\pi\omega)^2\right]\left[\beta^2 + (2\pi\omega)^2\right]} \tag{A.3}$$

represents the frequency gain function. Using (A.2) with $J = \mathrm{rhs}(20)$, the power spectrum of the CFM can be readily obtained. Figure A.1 illustrates (A.2) for $\alpha = 1/\tau^{(\mathrm{mem})}$, $\beta = 1/\tau^{(\mathrm{syn})}$ for a typical parameter setting used in our numerical simulations.

Note that the MFM describes a parametrically excited system [1] and as such its non-linearity prohibits to follow the same procedure as with the CFM and (A.1 & A.2) remain valid only for the CFM.

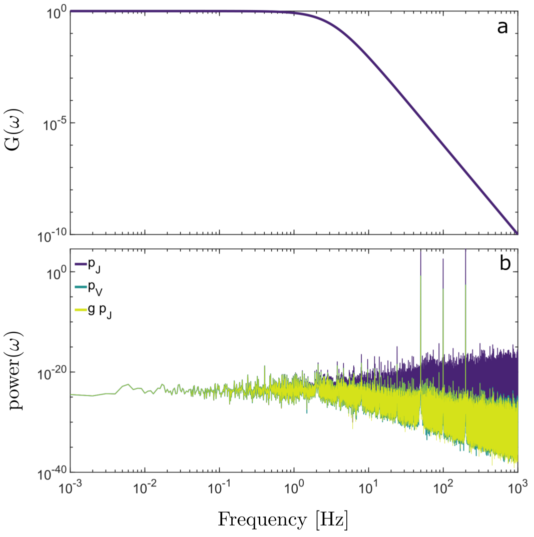

FIG. A.1. (a) Gain function $G(\omega)$ for the particular case of $\tau^{(\mathrm{mem})} = 20$ ms and $\tau^{(\mathrm{syn})} = 5$ ms. (b) Power spectrum of the LIF mean-field P_V in green, and that of the input p_J in blue, together with $G \cdot P_J$ in yellow. $G \cdot P_J$ is displayed with thinner lines on top of P_V to improve legibility.

Partial observations

Linking theoretical notes – like the current one – to experimental findings can be challenge but, as said, this should be consider the litmus test stressing their potential impact. If we consider our LIF model as a 'real' neuronal network, its experimental investigations may be limited in that not all (types of) neurons can be assessed. For instance, in contrast to the recording of local field potentials, encephalographic recording are believed to primarily pick up population activity of excitatory cells (e.g., EEG is considered to record post-synaptic potential changes in pyramidal cells that are spatially aligned and radially oriented in the neocortex). To mimic this, we re-analyzed our simulation by selecting from the full LIF-network the excitatory units and (1) used only them to estimate the LIF-network's mean field potential, i.e., $V \to V_{\mathrm{ex}}$, and (2) included them as input to the two dif-

ferent neural mass models, CFM and MFM, e.g., in(20) the sum over σ only contained $\sigma = E$. Then, we conducted the same comparisons as in the main part of the paper.

The corresponding results are summarised in Figure A.2. As expected, the limitation to only the excitatory population did not yield any qualitative changes when compared to the findings for the entire network (cf.(5) and (4)); note that we also tested this for only the inhibitory units, which in fact display very similar results. In brief, only in the close vicinity of the transition for the de-synchronised to the synchronised state, the mean field models showed proper resemblance with the dynamics of the underlying LIF-network. Yet, when looking at the CFM spectra, we could also identify good agreement when focusing on very small values of λ, which can be attributed to the fact that in this region the number of active neurons is particularly small. Having encephalographic recordings in mind, however, such a scenario is highly unlikely since, as said, pyramidal cells are believed to be the main contributor to the recorded signals and they are known to be primarily excitatory.

Taken together, our conclusions are not limited to experimental recordings of the full network and seemingly apply to partial assessments of the underlying neuronal population.

[1] Alan Champneys. Dynamics of parametric excitation. In Robert A. Meyers, editor, *Encyclopedia of Complexity and Systems Science*, pages 1–31, New York, NY, 2009. Springer New York.

[2] Ronald A. Fisher. Frequency distribution of the values of the correlation coefficient in samples from an indefinitely large population. *Biometrika*, 10(4):507–521, 1915.

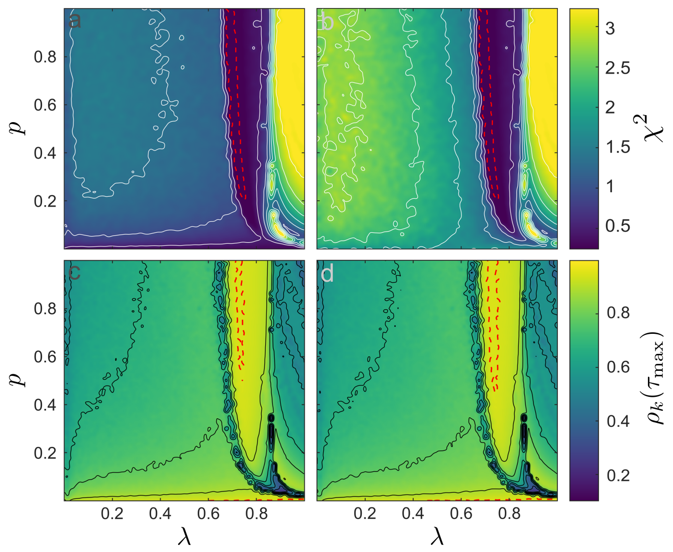

FIG. A.2. χ^2-statistic and correlation coefficients for the partial observation of only the excitatory units. Panels (a-b) display the $\chi^2(\cdot,\cdot)$ between the power spectra in the $\{p,\lambda\}$ space (10^5 values). (a) χ^2 between the excitatory units of the LIF network and the CFM feeded only with the excitatory units from the LIF model, $\chi^2(P^E_{\mathrm{LIF}}, P^E_{\mathrm{CFM}})$. (b) χ^2 between the excitatory units only from the LIF network and the MFM with only the excitatory units from the network as input, $\chi^2(P^E_{\mathrm{LIF}}, P^E_{\mathrm{MFM}})$. Again, we added several contour lines in white to improve legibility. Conform, e.g., Fig. (4) the dashed-red lines indicate the boundaries of significance regions ($\alpha = 0.01$ using the χ^2 distribution): inside the small region encircle by the dashed-red line, the CFM/MFM spectra were not significantly different from the LIF network spectrum. In panels (c-d) the corresponding correlation coefficients $\rho_k(\tau_{\max})$ between the mean field computed over only the excitatory units of the LIF model and (c) CFM and (d) MFM are shown; both computed with only the excitatory units from the LIF as input. The red-dashed lines in panels (c-d) indicate boundaries of significance with $\alpha = 0.01$ obtained by applying the Fisher transformation to the correlation values [2]. Inside the area defined by the red-dashed line in the synchronized region and the small area in the asynchronous region where $p \to 0$, the time series of the two neural mass models were not significantly different than the LIF mean field.
